# Deprivation and adverse outcomes from cardiac arrest^☆^

**DOI:** 10.1016/j.resplu.2025.100895

**Published:** 2025-02-05

**Authors:** Adam J. Boulton, Terry Brown

**Affiliations:** Warwick Clinical Trials Unit, Warwick Medical School, University of Warwick, Coventry CV7 4AL, UK; Out-of-Hospital Cardiac Arrest Outcome (OHCAO) Project, Clinical Trials Unit, Warwick Medical School, University of Warwick, Gibbet Hill Road, Coventry CV4 7AL, UK; Applied Research Collaboration West Midlands (ARCWM), Clinical Trials Unit, Warwick Medical School, University of Warwick, Gibbet Hill Road, Coventry CV4 7AL, UK

Health inequalities relate to systemic, avoidable, and unjust differences in health outcomes between groups of people.^1^, ^2^ These disparities can occur between entire populations, within subgroups of a population, or along a gradient based on social position.^1^ Differences in the conditions in which people are born, grow, live, work and age affect the opportunities for good health.^2^, ^3^ Addressing health inequalities has become a priority for governments and healthcare systems worldwide.^2^, ^4^, ^5^ There is growing international evidence of health inequalities in resuscitation, with people of lower socio-economic status and minority ethnicities having a higher incidence of cardiac arrest, disparities in care throughout the chain of survival, and poorer outcomes.^6^ Health inequalities in resuscitation are a key focus for Emergency Medical Services (EMS) and resuscitation organisations.^7^, ^8^, ^9^

Inequalities in the chain of survival in United Kingdom (UK) practice have been demonstrated, including in provision of bystander cardiopulmonary resuscitation (CPR) and availability of public access defibrillators.^10^, ^11^, ^12^ However, there is a relative sparsity of UK research investigating the influence of ethnicity and socio-economic status on outcomes, particularly in comparison to the global literature.^13^, ^14^, ^15^, ^16^, ^17^ In this issue of Resuscitation Plus, Owen *et al.* make a valuable contribution by examining the association between neighbourhood deprivation and survival following out-of-hospital cardiac arrest.^18^ One semi-rural region in Southern England with a population of approximately 1.4 million is investigated using local ambulance service data from January 2019 to March 2023, capturing 4,183 adult non-traumatic out-of-hospital cardiac arrest (OHCA) patients. The patient’s home postcode was used to obtain an Index of Multiple Deprivation (IMD) decile, a comprehensive measure of neighbourhood socio-economic status used in England that combines seven domains. Univariable logistic regression modelling found no significant association between IMD decile and the odds of survival at 30-days. However, when age, sex, initial rhythm, and bystander CPR were adjusted for in a multivariable model, a significant relationship emerged, indicating that lower deprivation (higher IMD decile) was associated with an increased odd of survival (odds ratio 1.05, 95% CI 1.01–1.09). Further models incorporating non-linear relationships for continuous co-variables to improve model fit found similar associations between deprivation and survival.

The association between deprivation and poorer survival following OHCA is consistent with previous literature.^13^, ^14^ The largest UK study to date by Bijman *et al.* included patients in Scotland between 2011 and 2020.^17^ Similarly, the study population was adult non-traumatic OHCAs treated by the local ambulance service and 20,585 individual records were included. Data linkage between several ambulance service records and hospital care records to gather patient demographics and mortality resulted in only 1.6% of the population being excluded due to missing data in one or more variable. Scottish IMD quintiles were used, modelled as categorical variables. Both unadjusted and adjusted analyses by age, sex, and urban/rural index found lower socio-economic status was associated with a reduced chance of 30-day survival. The authors go further, assessing for effect modification by stratification, finding that survival amongst males < 45 years had the strongest association with deprivation, whereas no association was found amongst either males or females > 80 years. A sensitivity analysis using incident location deprivation rather than patients home produced similar results. By contrast, Owen *et al.* imputed incident location when the home postcode was missing in 7% of cases, but it is worth noting that the incident and home postcode were the same in 70% of non-missing cases. Differences in measures of deprivation and modelling of these variables within the analyses between Owen *et al.* and Bijman *et al.* make comparing magnitude of effect estimate problematic. This is a common occurrence across the international literature with heterogeneity in the measures of deprivation, some of which may be society specific.^17^, ^19^ Regardless, there is a consistent association between increased deprivation and poorer survival following OHCA across a range of studies using varying deprivation measures and statistical approaches.^13^, ^14^, ^16^, ^17^, ^18^, ^19^

There are plausible mechanisms driving these differences in outcome. Studies examining the individual links in the chain of survival have found that health inequalities exist at each stage. It is therefore predictable that disparities in the pathway of care culminate in variations in outcome.^20^ Differences in provision of bystander CPR and distribution of public access defibrillators have been demonstrated in multiple systems, including the UK.^10^, ^11^, ^12^, ^13^, ^21^, ^22^, ^23^ Further, barriers to calling the EMS agencies, difficulties in the emergency call and delays in recognising cardiac arrest and beginning CPR, as well as access to resuscitation training also appear to contribute.^6^, ^24^, ^25^, ^26^, ^27^ Disparities have also been demonstrated in post resuscitation care, including coronary angiography and intervention.^28^, ^29^ Studies utilising mediation analyses help to quantity the direct effect of socio-economic status on outcome, as well as the indirect effect through intermediate variables. A systematic review of ten such studies found that initial rhythm was the most important mediator, followed by EMS response time and bystander CPR.^30^ One of the included recent studies from South Korea illustrates the power of high quality registry data in investigating health inequalities. Choi *et al.* studied over 120,000 OHCA patients and captured variables related to all links in the chain of survival, including post resuscitation in-hospital care.^16^ Further, linkage with the national health insurance database allowed individual socio-economic status to be captured as opposed to neighbourhood. Again, OHCA patients of lower socio-economic status had poorer survival. Mediation analysis found that witnessed status, bystander CPR, initial rhythm, and emergency department type were the key mediators. Among patients who survived to hospital admission, coronary angiography and targeted temperature management were additionally found to contribute to disparities in outcome. This kind of analysis is valuable in highlighting the modifiable factors that should be addressed to tackle health inequalities and rely on high quality data sources.

The paper by Owen *et al.* offers a valuable starting point for future research to build upon in England. The key limitations relate to data quality, which the comparisons with Bijman *et al.* and Choi *et al.* underline.^16^, ^17^ A retrospective review of local ambulance service records was performed and deficiencies in data quality were encountered in several variables, most notably in witnessed status. This prevented identification of the EMS witnessed cohort and may have contributed to the complexities in response time modelling. Witnessed status influences provision of bystander CPR, and so the interpretation of bystander CPR without witnessed status could be misleading. It also prevents identification of the Utstein Comparator Group, a valuable comparative subgroup. Prospective national registries offer a potential solution to this challenge. For example, the large national registry from multiple data sources used by Choi *et al.* not only had high data quality throughout collected variables with low missingness, but was also able to include a broader set of prehospital factors, as well as post-resuscitation in-hospital variables too. It is commendable that Owen *et al.* have used local clinical records to investigate a pertinent issue in resuscitation science, demonstrating the value of utilising electronic health records in research. It also highlights the challenges of these data sources and underlines the need for future research to utilise improved data source quality, enabling more robust and insightful analyses. This will be essential to better understanding patterns, identifying modifiable factors, and driving interventions to tackle health inequalities in resuscitation.

## Declaration of competing interest

The authors declare the following financial interests/personal relationships which may be considered as potential competing interests: Adam Boulton (Doctoral Research Fellow, NIHR303023) is funded by the NIHR. Terry Brown is supported by funding from the British Heart Foundation and Resuscitation Council UK (OHCAO Project) and NIHR (ARCWM). He has also received funding for research from NIHR HSDR. The views expressed in this publication are those of the authors and not necessarily those of the NIHR, NHS or the UK Department of Health and Social Care.
